# Supplementary material for: Amphiphilic Polymer Conetwork Gel Films Based on Tetra-Poly(ethylene Glycol) and Tetra-Poly(ε-Caprolactone)
Source: Polymers (Basel). 2022 Jun 23;14(13):2555. doi: 10.3390/polym14132555 (PMC9269314; doi:10.3390/polym14132555)
Supplement: Supplementary file 1 [file polymers-14-02555-s001.zip › Supplementary Material.pdf]

Supplementary Material

Amphiphilic Polymer Conetwork Gel Films Based on tetra-poly(ethylene glycol) and tetra-poly(ε-caprolactone)

Kevin Hagmann [1], Carolin Bunk [2,3], Frank Böhme [2] and Regine von Klitzing [1,*]

[1] Institute for Condensed Matter Physics, Technische Universität Darmstadt, Hochschulstr. 8, D-64289 Darmstadt, Germany; kevin.hagmann@pkm.tu-darmstadt.de
[2] Leibniz-Institut für Polymerforschung, Dresden e.V, Hohe Str. 6, D-01069 Dresden, Germany; bunk@ipfdd.de (C.B.); boehme@ipfdd.de (F.B.)
[3] Organic Chemistry of Polymers, Technische Universität Dresden, D-01062 Dresden, Germany
* Correspondence: klitzing@smi.tu-darmstadt.de; Tel.: +49-06151-16-24506.

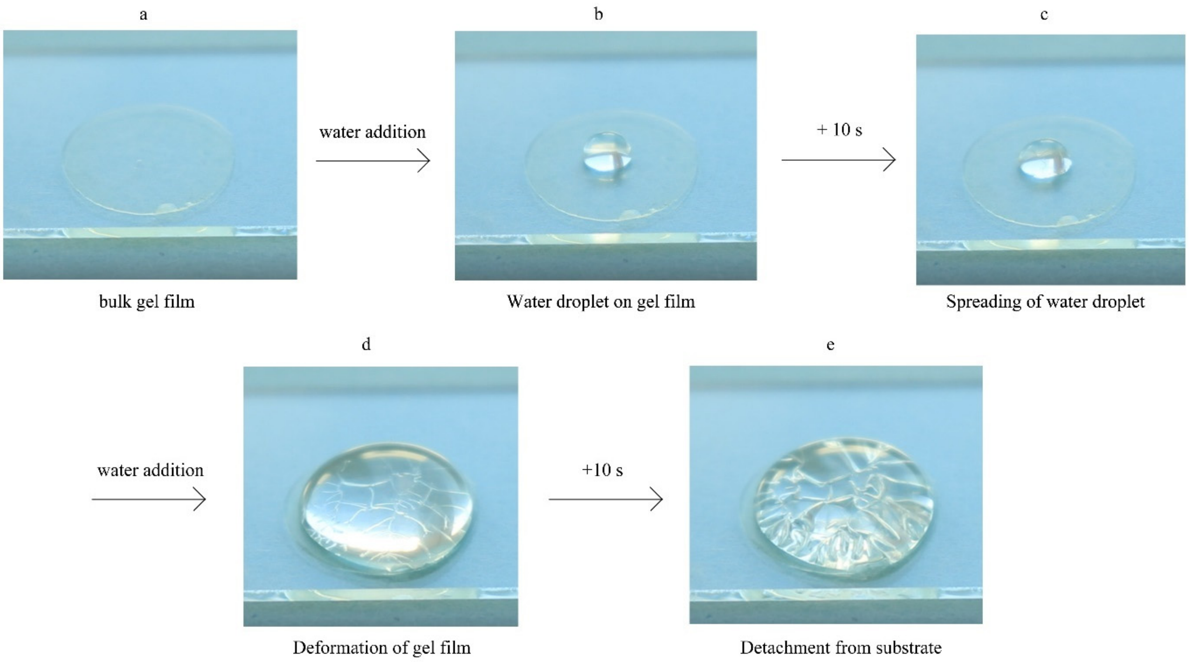

Figure S1: Swelling behavior of gel film in water. a) Dried bulk gel film, b) Pipetting of water droplet onto gel film, c) After a few seconds the water droplet noticeably penetrates the network, the water droplet spreads/contact angle decreases. d) The swelling and deformation of the gel film is better visualized through the addition of additional water. e) The gel film fully deforms and detaches from the glass substrate

Video S1: Swelling behavior of bulk gel film in water

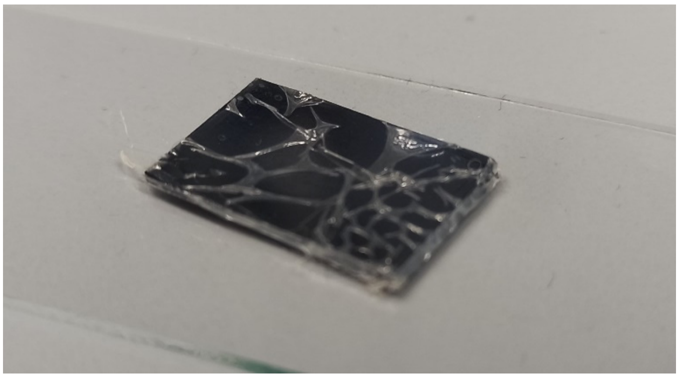

Figure S2: Deformation of ACN thin gel film (thickness ~ μm) and detachment from silicon substrate after the addition of water.
