# Supplementary figures and images for: Amphiphilic Polymer Conetwork Gel Films Based on Tetra-Poly(ethylene Glycol) and Tetra-Poly(ε-Caprolactone)
Source: Polymers (Basel). 2022 Jun 23;14(13):2555. doi: 10.3390/polym14132555 (PMC9269314; doi:10.3390/polym14132555)

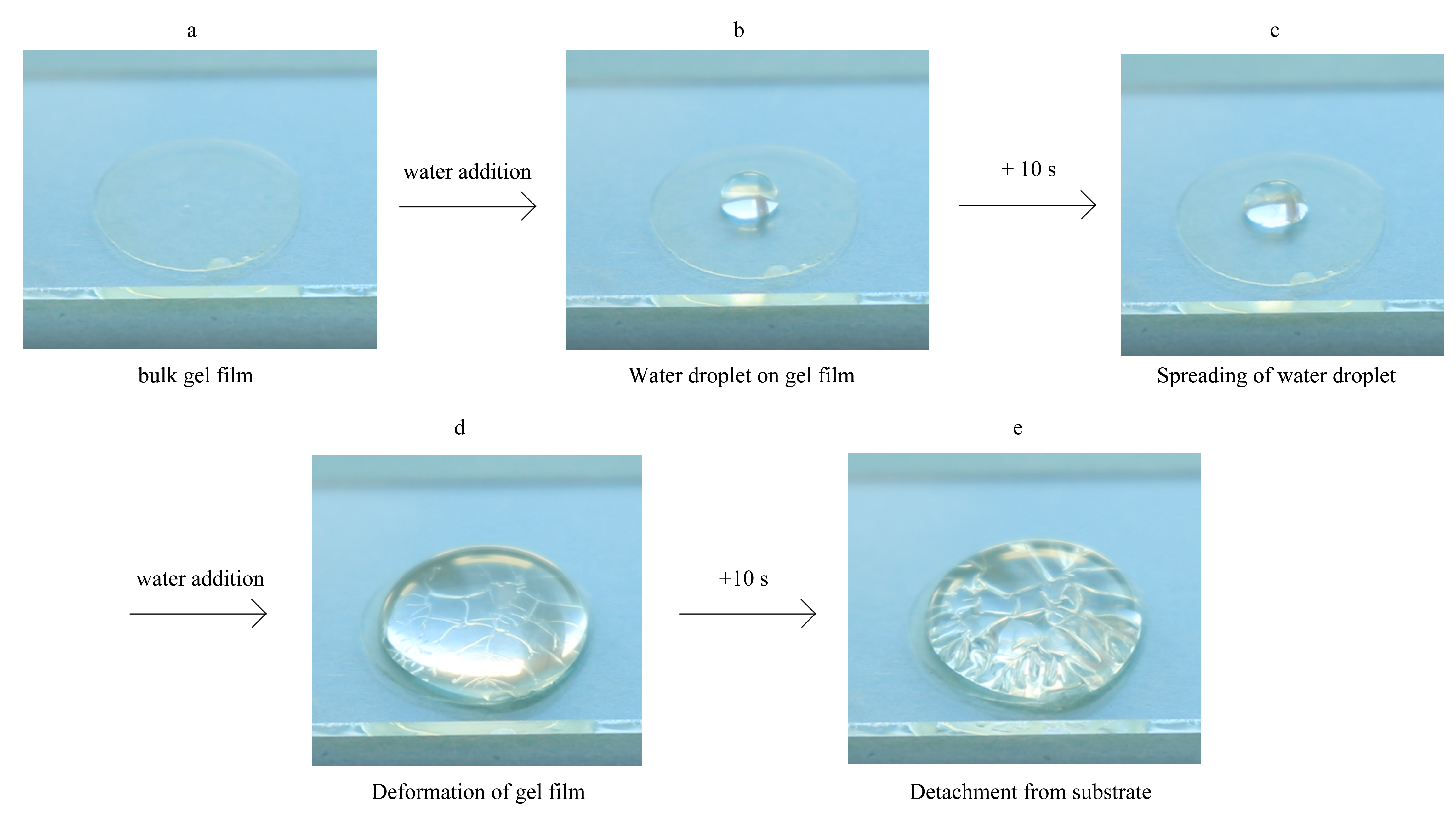

Supplement: Supplementary file 1 [file polymers-14-02555-s001.zip › Figure S1.jpg]

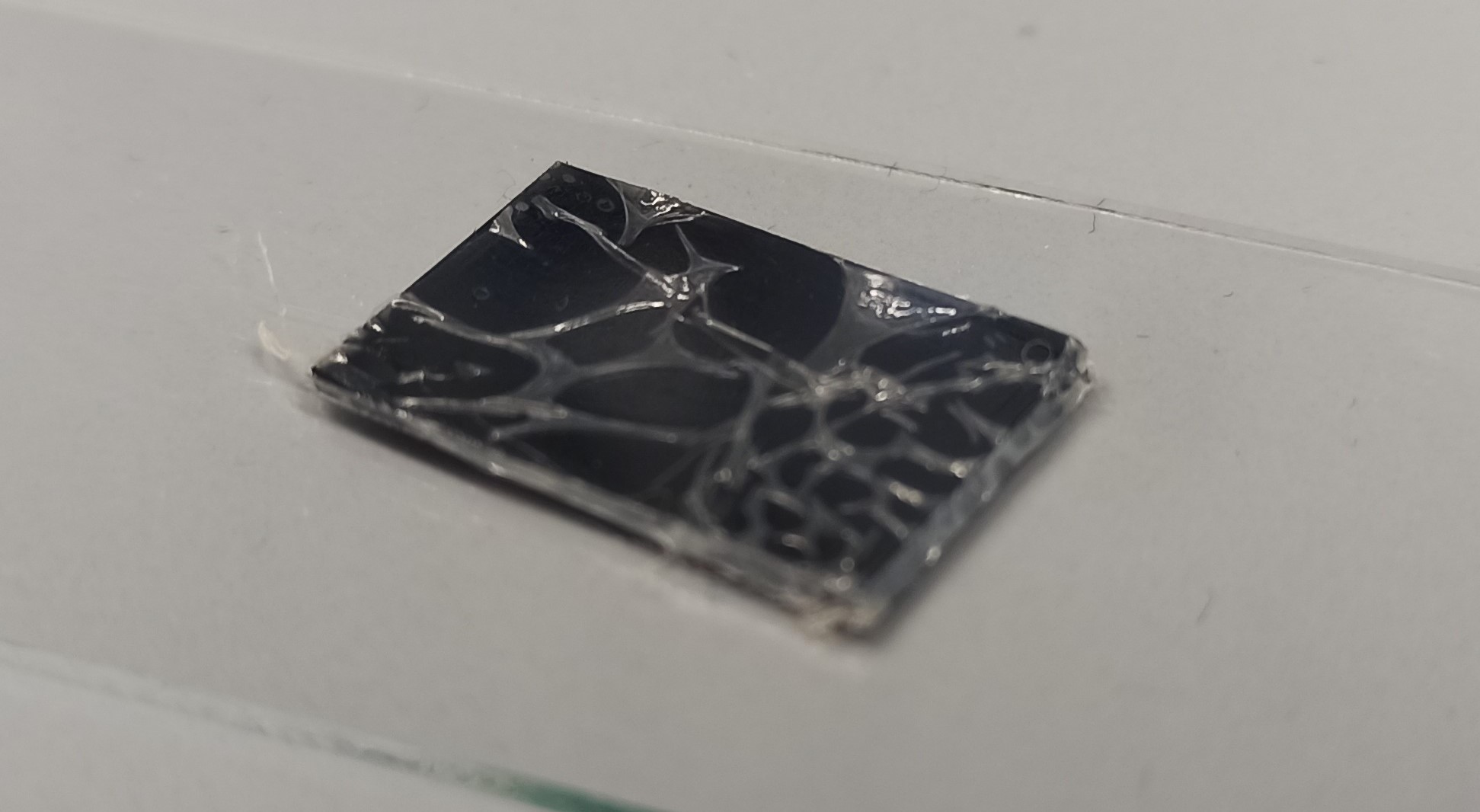

Supplement: Supplementary file 1 [file polymers-14-02555-s001.zip › Figure S2.jpg]
